# Crepitus following a dog bite

**DOI:** 10.1002/ccr3.7520

**Published:** 2023-06-09

**Authors:** Kenbon Seyoum, Telila Mesfin, Gosaye Debele, Kebebe Bekele, Bekele Chimdesa, Debela Kebe

**Affiliations:** ^1^ Department of Midwifery Goba General Hospital Goba Ethiopia; ^2^ School of Medicine Goba General Hospital Goba Ethiopia; ^3^ Department of surgery Goba General Hospital Goba Ethiopia

**Keywords:** crepitus, dog bite, wound care

## Abstract

Crepitus following an animal bite is a rare case. We report a case of a 20‐year‐old man who presented to the surgical emergency department 1 h after being bitten by a medium‐sized pet dog.

## Graphical abstract

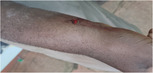

## 1 INTRODUCTION

Crepitus is a palpable or audible grating or crunching sensation produced by motion, and it is felt beneath the skin and in the joints.^1^, ^2^ It occurs when gas is introduced into a region of soft tissues where it is not ordinarily present.^2^ Anaerobic bacteria like clostridia, peptostreptococci, bacteroides, or aerobic coliform are typically to blame for gas‐forming illnesses on the surgical service.^3^

Animal bites are widespread around the world and could cause serious illness. In North America, there were two to 5 million animal bites every year, which result in 10,000 inpatient admissions and around 1% of visits to emergency rooms.^4^ Most victims of the animal bite were children,^5^ with children 5–9 having an incidence of 60.7% per 10,000.^6^ Worldwide, tens of millions of dog bites occur annually, and it accounts for 76%–94% of animal bites in low and middle‐income countries.^7^ It is the most common bite injury accounting for 80%–90% of presentations.^6^ Crepitus after a dog bite raises the possibility of a gas‐forming infection, which needs prompt medical and surgical intervention.^8^ About 1% of all visits to emergency rooms are for bite‐related injuries, the majority of which are caused by dogs. Exotic animals' oral microbiota is reflected in the bacteriology of their bite wounds.^9^

As to the knowledge of the authors, there is only one case reported on Crepitus after a dog bite, and thus why this case report is also considered to be reported concerning this rare case after it is seen incidentally.

## 2 CASE PRESENTATION

Giving attention to the presence of crepitus following a dog bite will help clinicians to identify and treat crepitus related health problem. Most of the time, the clinicians pay attention only to the wound. This report will raise awareness about crepitus after a dog bite. A 20‐year‐old Ethiopian man presented to our surgical emergency department 1 h after a dog bite. The dog had cut the rope 2 days before. The dog, which belonged to the victim's family, bit the man after provoking him. The bite injury was on his right arm, the bite site was wounded, and he was also bleeding and felt pain. The dog has not taken any vaccination before and has been followed for any behavioral change. Otherwise, the patient has no history of diabetes, retroviral infection, cancer or has not taken steroid drugs.

**FIGURE 1:**
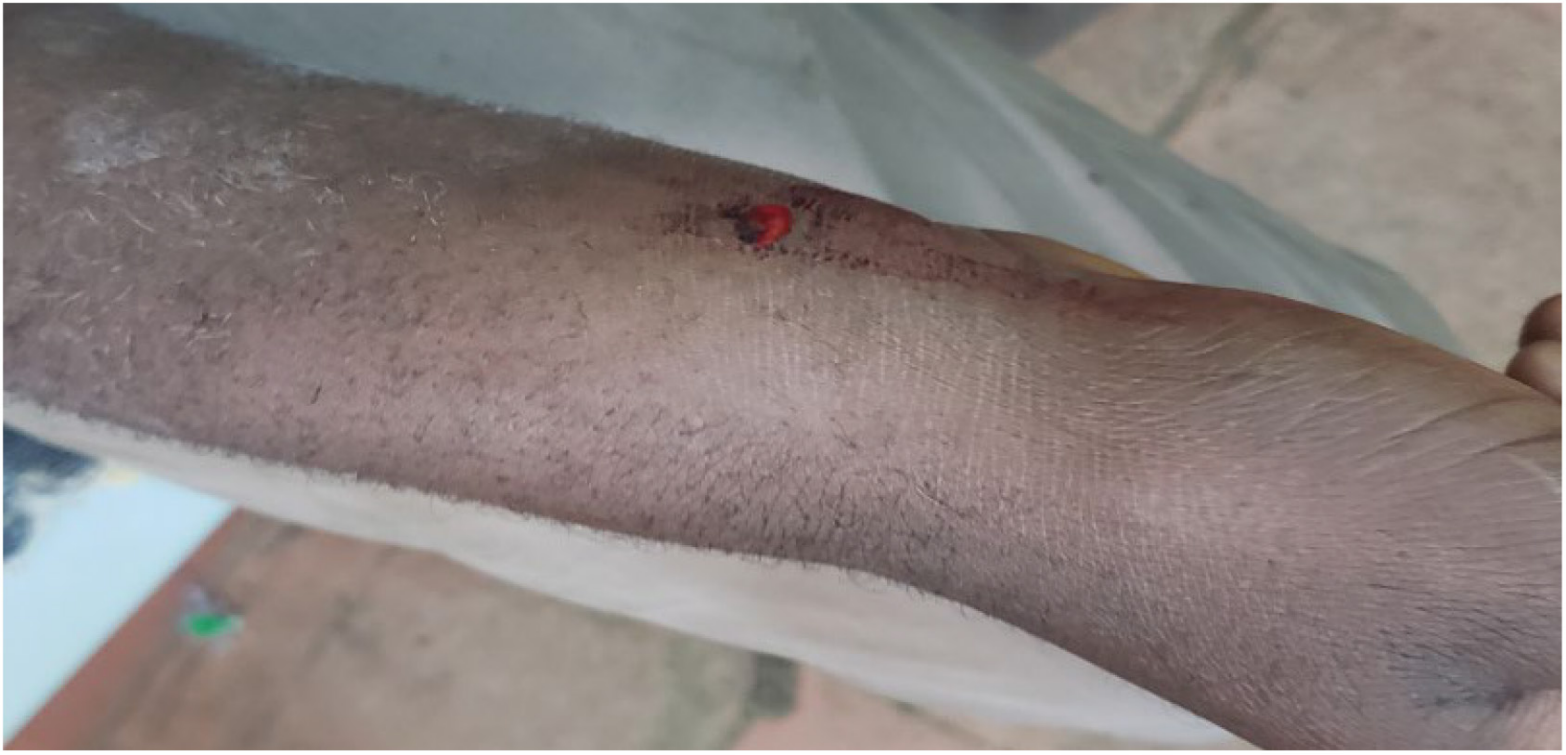
Puncture wound on the distal lateral aspect of the right forearm with swelling.

On examination, there was around 0.5 × 0.5 cm puncture wound over the lateral aspect of the right distal forearm and 2 × 1 cm over the posterior aspect at the same level. There was crepitus to the level of the elbow (Figure 1). The wound was clean with bleeding from the site, no surrounding erythema, and odorless. The right forearm was swollen to the level of the elbow. There was crepitus which was consistent with surgical emphysema on palpation (Figure 1). There was the pain‐free movement of fingers and wrist with intact distal neurovascular structure. He appeared systemically well with normal vital signs (Blood pressure was 100/70 mmHg, pulse rate 80 beats/min, temperature 36.7°C, respiratory rate 18 breaths/min).

**FIGURE 2:**
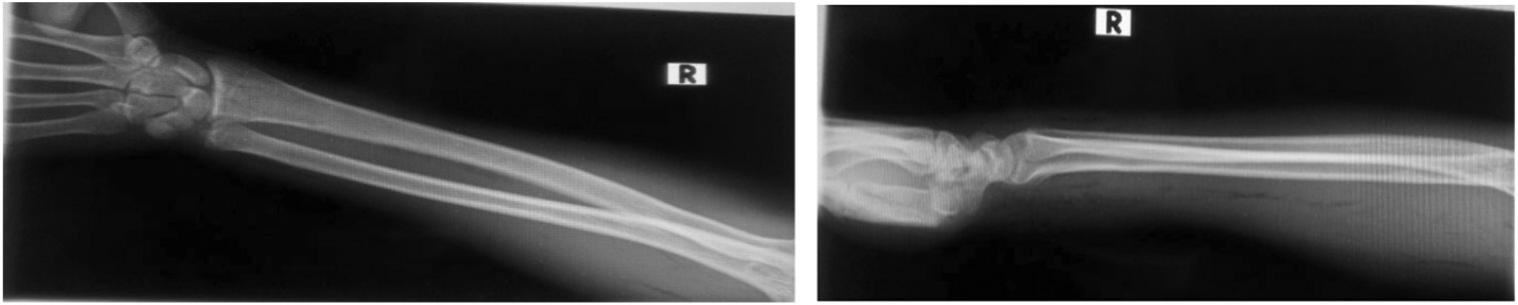
Radiographs demonstrating subcutaneous gas in the forearm.

On laboratory investigations: His white cells’ count on admission was 8700/µL, and blood culture was not done due to the lack of necessary medium. His random blood sugar was 162 g/dL and non‐reactive for retroviral infection (HIV). Right forearm X‐ray demonstrated subcutaneous gas (Figure 2).

**FIGURE 3:**
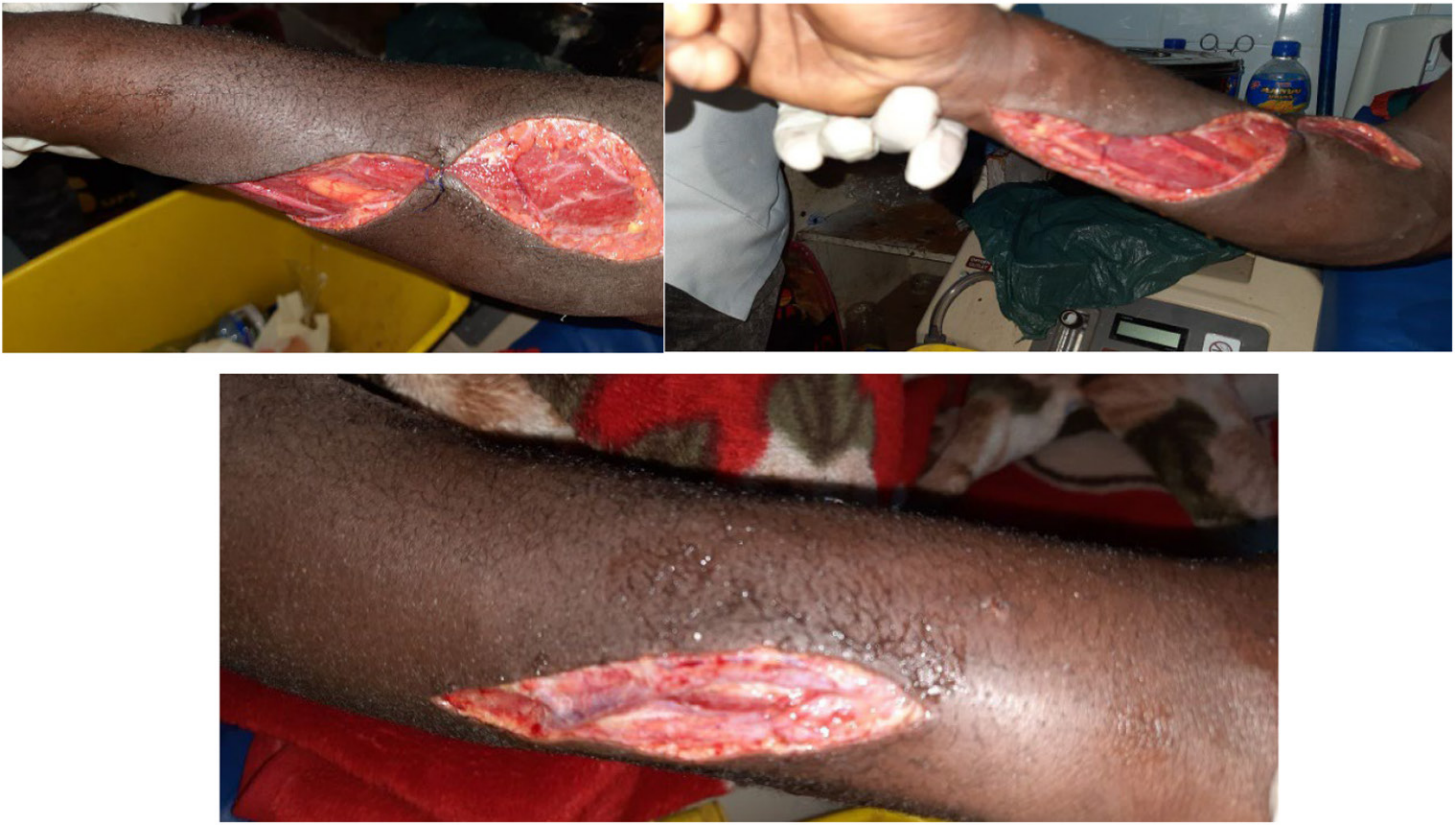
Right forearm after the fasciotomy was done.

He was admitted under the care of an orthopedics and trauma surgeon with the suspected diagnosis of necrotizing fasciitis of the right forearm. The patient was taken to the major operation room after written informed consent was taken from him. Under possible aseptic technique and axillary block, the right extremity was prepared and draped. The incision was made from the wrist to the elbow joint, the fascia was opened, and there was minimal muscle color change upon entrance which regained normal color soon. The right forearm fasciotomy was done (incision made anteriorly from wrist to elbow joint and posteriorly just above wrist joint); the wound was washed with normal saline and hydrogen peroxide and left open for wound care (Figure 3).

He has been given tetanus prophylaxis, intravenous antibiotics (ceftriaxone, metronidazole), and analgesia and kept in the hospital for wound care and observation. He has been given daily wound care in the hospital with normal saline and hydrogen peroxide.

Postoperatively, he was stable with the normal vital sign and repeated white blood cells were 7380/µL.

**FIGURE 4:**
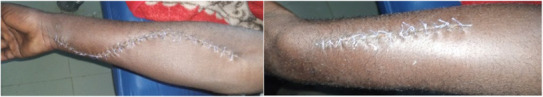
After fasciotomy wound closure.

After the swelling subsided, the absence of signs of infection and crepitus was checked and the surgical wound closed after 3 days of operation (Figure 4). The patient was sent home with oral antibiotics (cephalexin 500 mg PO BID for 5 days and metronidazole 500 mg PO TID for 5 days) with a short appointment for follow‐up.

## 3 DISCUSSION

The first case of crepitus following a dog bite was reported in 1998.^8^ The current case would be the second, making it a rare case.

Patients who presented early are less likely to have established infection than those who presented late. The median time to the first signs and symptoms of infection following a dog bite is 24 h.^10^ In the current case, signs and symptoms such as swelling and tenderness were started within an hour which was faster than the median time stated above. Even if clinical manifestations of bite wound infections may include fever, erythema, swelling, tenderness, purulent drainage, and lymphangitis,^6^, ^10^ the patient has no fever, erythema, and purulent discharge which may be due to his earlier presentation.

The majority of dog bite wounds are mild and just need to be treated locally. Inexpensive penicillins or cephalosporins are sufficient for early treatment if antimicrobial medicines are required.^11^ However, the formation of crepitus with extensive swelling necessitates fasciotomy to be done beyond local wound care in the current victim, even though the wound was not such extensive.

Antibiotics taken as a preventative measure lower the risk of infection in people with dog bite wounds.^12^ In our patient, intravenous antibiotics were given to cover both aerobics and anaerobic bacteria, because etiologies were not identified. In addition, tetanus prophylaxis was also given to prevent tetanus infection.

## 4 CONCLUSION

Patients with dog bites may develop rare complications like crepitus. Crepitus formation may not only be experienced in patients with common risk factors; hence, better to follow for all patients with dog bites and early evaluation and referral should be considered. The thorough evaluation of such patients should be timely and should not target only the wound site.

## AUTHOR CONTRIBUTIONS

**Kenbon Seyoum:** Project administration; writing – original draft; writing – review and editing. **Telila Mesfin:** Data curation; visualization; writing – review and editing. **Gosaye Debele:** Conceptualization; investigation; resources. **Kebebe Bekele:** Investigation; validation; visualization. **Bekele Chimdesa:** Investigation; resources; validation. **Debela Kebe:** Resources; validation; visualization; writing – review and editing.

## CONSENT

Written informed consent was obtained from the patient to publish this report in accordance with the journal's patient consent policy.

## Data Availability

Data are available from the corresponding author upon reasonable request.
